# Common cancer treatments targeting DNA double strand breaks affect long-term memory and relate to immediate early gene expression in a sex-dependent manner

**DOI:** 10.18632/oncotarget.28180

**Published:** 2022-01-24

**Authors:** Sydney Weber Boutros, Destine Krenik, Sarah Holden, Vivek K. Unni, Jacob Raber

**Affiliations:** ^1^Department of Behavioral Neuroscience, Oregon Health and Science University, Portland, OR 97239, USA; ^2^Department of Neurology, Oregon Health and Science University, Portland, OR 97239, USA; ^3^Jungers Center for Neurosciences Research, Oregon Health and Science University, Portland, OR 97239, USA; ^4^OHSU Parkinson Center, Oregon Health and Science University, Portland, OR 97239, USA; ^5^Department of Psychiatry, Oregon Health and Science University, Portland, OR 97239, USA; ^6^Department of Radiation Medicine, Oregon Health and Science University, Portland, OR 97239, USA; ^7^Division of Neuroscience, The Oregon National Primate Research Center, Oregon Health and Science University, Portland, OR 97239, USA

**Keywords:** amifostine, etoposide, double strand breaks, memory, sex

## Abstract

DNA double strand breaks (DSBs) have been highly studied in the context of cancers, as DSBs can lead to apoptosis or tumorigenesis. Several pharmaceuticals are widely used to target DSBs during cancer therapy. Amifostine (WR-2721) and etoposide are two commonly used drugs: amifostine reduces DSBs, whereas etoposide increases DSBs. Recently, a novel role for DSBs in immediate early gene expression, learning, and memory has been suggested. Neither amifostine nor etoposide have been assessed for their effects on learning and memory without confounding factors. Moreover, sex-dependent effects of these drugs have not been reported. We administered amifostine or etoposide to 3–4-month-old male and female C57Bl/6J mice before or after training in fear conditioning and assessed learning, memory, and immediate early genes. We observed sex-dependent baseline and drug-induced differences, with females expressing higher cFos and FosB levels than males. These were affected by both amifostine and etoposide. Post-training injections of amifostine affected long-term contextual fear memory; etoposide affected contextual and cued fear memory. These data support the hypothesis that DSBs contribute to learning and memory, and that these could play a part in cognitive side effects during common treatment regimens. The sex-dependent effects also highlight an important factor when considering treatment plans.

## INTRODUCTION

Unrepaired DNA double strand breaks (DSBs) are signals for apoptosis [1] and drugs to induce DSBs or protect non-tumorous tissue from DSBs have been developed to treat cancers. Amifostine (WR-2721) is metabolized into the active agent WR-1065, and protects cells from DSBs by scavenging free radicals, inducing cellular anoxia, and condensing DNA [2–4]. Conversely, etoposide induces DSBs by stabilizing the complex of covalently-bound topoisomerase-II beta to cleaved DNA [5]. Recent evidence suggests that DSBs may induce immediate-early gene (IEG) expression [6, 7].

The precisely timed expression of IEGs is essential for learning and memory [8]. The *fos* family of genes, including fos proto-oncogene (cFos) and FosB, are IEGs important in fear learning and memory [9]. cFos is transiently expressed, with low basal levels that rapidly increase upon stimulation, and return to baseline levels within hours [10]. FosB has similarly low basal levels, though the truncated form (ΔFosB) accumulates over time and persists for weeks following a stimulus [11]. Hippocampal cFos and ΔFosB are essential for contextual learning and hippocampal synaptic plasticity [12, 13]. However, the mechanism underlying the rapid expression of IEGs is unclear.

Following physiological stimulation, DSBs were detected on promoter regions of IEGs, including *fos* and *fosB*, leading to their up-regulation [7]. Transient increases in the DSB repair marker γH2Ax were seen in relevant brain regions in mice following contextual learning [6, 7], and inhibition of DSB repair in the mouse prelimbic area prolonged IEG expression and impaired long-term memory [14]. Thus, DSB induction and timely repair may facilitate IEG transcription and learning. Relatedly, physiological DNA-damaging agents, such as reactive oxygen species (ROS) and superoxide, might contribute to learning and memory. Increasing and inhibiting activity of Nicotinamide adenine dinucleotide phosphate (NADPH) oxidase, which creates ROS and superoxide, impairs learning and memory [15]. NADPH is a target for cancer treatments, as NADPH oxidases contribute to chronic gastrointestinal inflammation and cancer [16], and targeting NADPH homeostasis might reduce tumors [17].

Despite their wide clinical use, there is little information about how amifostine and etoposide affect learning and memory. Etoposide may modulate learning and memory by inducing IEG expression [7], though prolonged IEG expression and impaired long-term memory were seen following microinjections of etoposide in the mouse prelimbic area [14]. Assessment of hippocampal volume in pediatric brain cancer survivors indicated smaller hippocampal volume following chemotherapy treatment, which included etoposide and amifostine, that corresponded to verbal memory performance [18]. As etoposide and amifostine were used in combination with other chemotherapy agents, it is impossible to conclude which effects were caused by these specific drugs. To the best of our knowledge, investigations into the effects of amifostine alone have not been reported, nor have there been reports on timing of administration of these drugs related to learning and memory. Timing is important to consider, as irradiation with gamma rays or X-rays (known to induce DSBs) after fear learning increased long-term fear memory in mice [19], which directly contrasts to impaired memory when mice are irradiated prior to fear learning [20]. Preclinical research into sex-dependent effects of amifostine and etoposide is also lacking. In cancer patients, women clear plasma amifostine faster than men [21]. Etoposide also has a lower half-life in females, though clearance is similar between the sexes [22].

In this study, we investigated the effects of amifostine and etoposide on hippocampus-dependent and -independent fear conditioning [23] and IEG expression in male and female C57Bl/6J mice.

## RESULTS

### Pre- and post-training injections of 107 mg/kg of amifostine increase long-term contextual, but not cued, freezing in male mice

**Figure 1:**
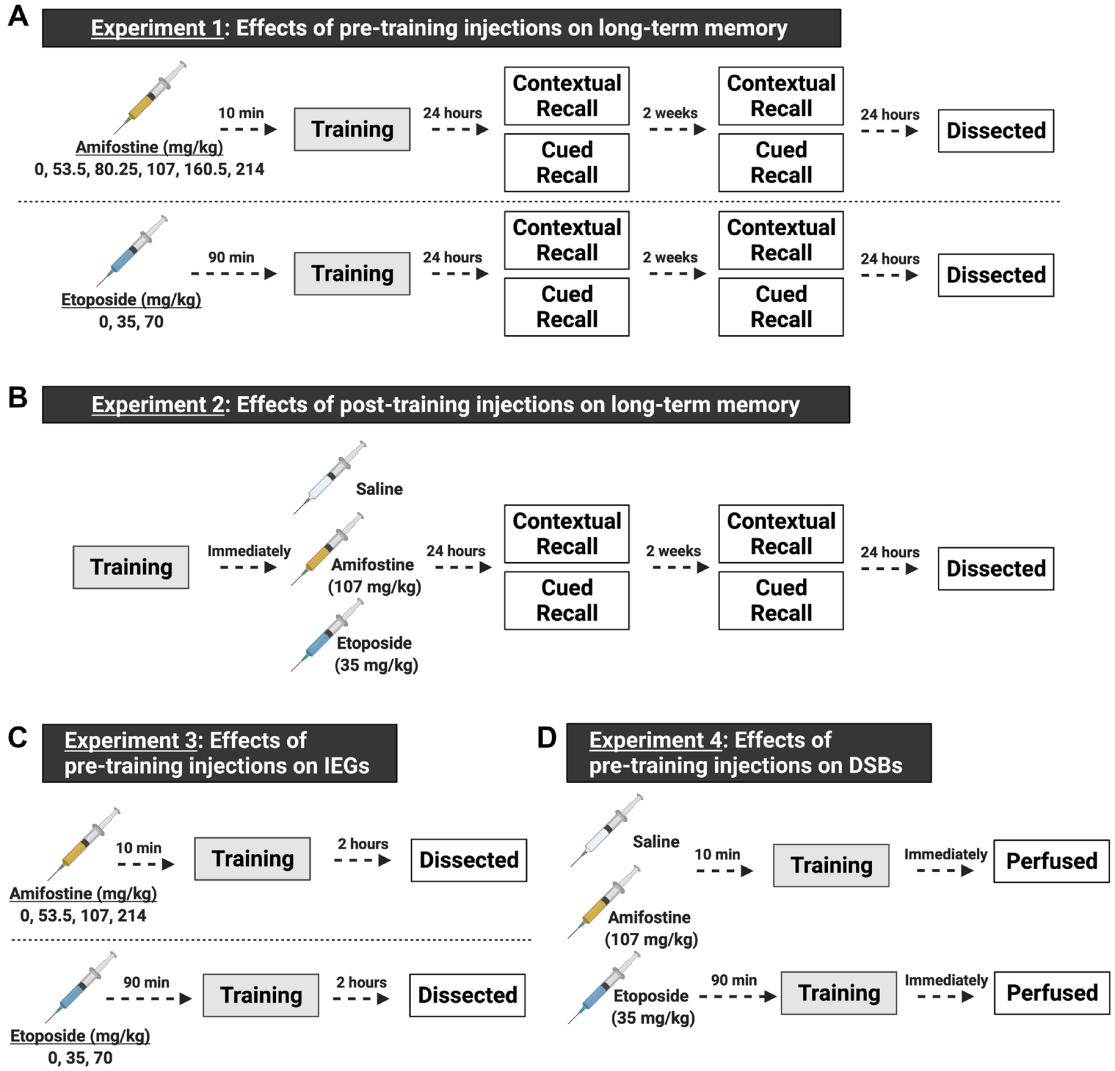
Schematic of the experimental design, made with https://biorender.com. Both male and female C57Bl/6J mice were used in all experiments. (**A**) Experiments for understanding the effects of pre-training injections on learning and memory. In experiment 1, mice received a single *i.p.* injection prior to being trained in fear conditioning. We tested 6 doses (including saline, *n* = 8–12 mice/sex/dose) of amifostine administered 10 min before fear training, or 3 doses (including saline, *n* = 8–9 mice/sex/dose) of etoposide administered 90 min before fear training. Twenty-four hours and 2 weeks later, mice underwent contextual and cued recall tests. One day after the last recall test, mice were euthanized, and brain tissue dissected. (**B**) Experiments for understanding the effects of post-training injections on learning and memory. In experiment 2, mice were trained in fear conditioning and immediately received an *i.p.* injection of saline, 107 mg/kg amifostine, or 35 mg/kg of etoposide upon completion (*n* = 7 mice/sex/dose). Doses were picked based on the results of experiment 1. Mice underwent the same timeline for recall tests as in experiment 1. (**C**) Experiments for understanding the effects of pre-training injections on immediate early genes. We tested 4 doses (including saline, *n* = 5 mice/sex/dose) of amifostine and 3 doses (including saline, *n* = 5 mice/sex/dose) of etoposide following the same timing as in experiment 1. Mice were euthanized 2 hours later to assess cFos expression. (**D**) Experiments for understanding the effects of pre-training injections on DSB formation. Based on results from the previous experiments, we injected mice with saline, 107 mg/kg amifostine, or 35 mg/kg of etoposide before training in fear conditioning (*n* = 3 mice/sex/dose). Immediately upon completion, mice were perfused to assess DSB formation.

**Figure 2:**
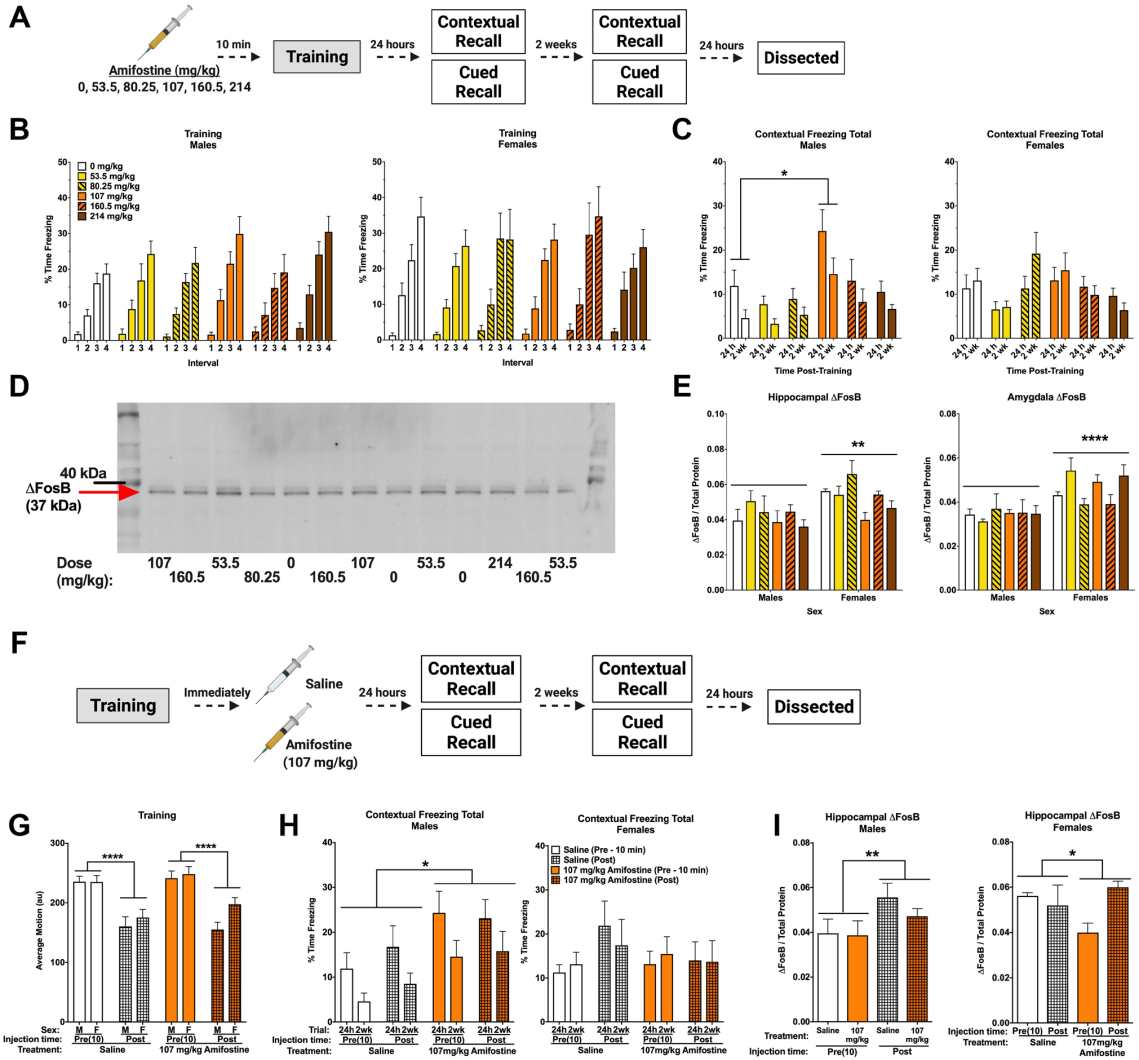
Pre- and post-training injections of amifostine lead to long-term increases in contextual freezing in males. (**A**) Experiment 1 design. (**B**) Percent time freezing during the inter-stimulus intervals on training day. All mice showed similar increases in time freezing, indicating fear learning. (**C**) Percent time freezing during the 5-min contextual recall tests done at 24 h and 2 weeks in males (*left*) and females (*right*). In males, a significant main effect of dose was detected (*F*(5,54) = 3.999, *p* = 0.004) with males injected with 107 mg/kg freezing more than 0 mg/kg (*p* = 0.017, Dunnett’s post hoc). In females, there was a significant main effect of dose detected (*F*(5,54) = 2.588, *p* = 0.036). Dunnett’s post hoc test did not indicate any significant differences compared to the 0 mg/kg group. (**D**) Representative Western blot for ΔFosB. (**E**) Quantification of hippocampal (*left*) and amygdala (*right*) ΔFosB levels. A 2-way ANOVA for hippocampal ΔFosB showed a significant main effect of sex (*F*(1,47) = 9.908, *p* = 0.0029) and a trend towards a main effect of dose (*F*(5,47) = 2.387, *p* = 0.0521). Sexes were split for analysis, and revealed a main effect of dose in females (*F*(5,23) = 3.202, *p* = 0.0244), with Dunnett’s post hoc test indicating a trend towards lower ΔFosB in the 107 mg/kg group compared to the 0 mg/kg group (*p* = 0.0569). There was no effect detected in males (*p* = 0.6366). A 2-way ANOVA for amygdala levels also indicated a significant main effect of sex (*F*(1,47) = 21.6, *p* < 0.0001), but no differences based on dose were detected. (**F**) Experiment 2 design. (**G**) Average baseline motion during the training session. Regardless of dose, pre-training injections increased average baseline motion (*F*(1,84) = 50.11, *p* < 0.001). (**H**) Percent time freezing during the 24 h and 2-week contextual recall tests in males (*left*) and females (*right*). Both pre- and post-training injections of 107 mg/kg of amifostine increased percent time freezing in male mice (*F*(1,29) = 6.741, *p* = 0.015). 107 mg/kg of amifostine did not affect percent time spent freezing in female mice (*p* = 0.611). (**I**) Hippocampal ΔFosB levels following pre- and post-training injections of saline or 107 mg/kg in males (*left*) and females (*right*). A 3-way ANOVA revealed a main effect of injection time (*F*(1,42) = 7.132, *p* = 0.001), dose (*F*(1,42) = 4.108, *p* = 0.049), and sex (*F*(1,42) = 8.265, *p* = 0.011), and a dose by time interaction (*F*(1,42) = 4.284, *p* = 0.045). Sexes were split for further analysis; an effect of time was found in males (*F*(1,21) = 4.724, *p* = 0.0413). In females, an effect of dose was found (*F*(1,21) = 4.601, *p* = 0.0438). Data show averages ± SEM. ^*^ *p* < 0.05; ^**^ *p* < 0.01; ^****^ *p* < 0.0001.

Figure 1 shows a timeline of all experiments. Mice received an intraperitoneal (*i.p.*) injection of saline, 53.5, 80.25, 107, 160.5, or 214 mg/kg amifostine 10 min prior to being trained in fear conditioning. Contextual and cued memory were tested 24 h and 2 weeks later (Figure 2A).

Amifostine had no effects on average motion or percent time freezing during fear training (Table 1). All groups increased freezing over the four tones (*p* < 0.001) and four inter-stimulus intervals (ISI; *p* < 0.001, Figure 2B), indicating similar fear learning. Females froze more than males during the ISIs (*p* = 0.046). Similarly, we detected no effects of amifostine on motion during the ISIs or tones.

**Table 1:** Fear conditioning motion and time freezing during training for mice in experiment 1 and 2 investigating the effects of amifostine

Dose (mg/kg)	Pre-Training Injections						Post-Training Injections	
	0	53.5	80.25	107	160.5	214	0	107
**Baseline Average Motion (au)**	247.06 ± 7.78	254.44 ± 7.74	242.39 ± 6.24	253.51 ± 11.26	211.07 ± 11.62	235.21 ± 8.88	*See Figure 2*	*See Figure 2*
**Shocks Average Motion (au)**	774.76 ± 33.18	837.52 ± 42.79	717.34 ± 43.3	826.64 ± 52.10	804.86 ± 32.83	801.35 ± 36.08	734.39 ± 42.17	735.37 ± 36.30
**Last Tone % Time Freezing**	29.57 ± 4.02	25.00 ± 2.90	30.34 ± 3.75	34.10 ± 4.74	31.71 ± 6.25	35.20 ± 4.67	35.94 ± 6.69	32.38 ± 4.29
**Last ISI % Time Freezing**	*See Figure 2*	*See Figure 2*	*See Figure 2*	*See Figure 2*	*See Figure 2*	*See Figure 2*	24.37 ± 4.28	26.87 ± 4.97

No differences were detected between sexes or doses. All data are presented as means ± SEM.

Analysis of total percent time freezing during the contextual recall tests revealed an effect of dose (*p* < 0.001) and sex (*p* = 0.046). Dunnett’s post hoc test indicated that mice injected with 107 mg/kg froze more than controls (*p* = 0.014). When we analyzed sexes separately, we found an effect of dose in both males (*p* = 0.004; Figure 2C, *left*) and females (*p* = 0.036; Figure 2C, *right*). Dunnett’s post hoc test indicated that males injected with 107 mg/kg froze more than saline-injected controls (*p* = 0.017), but this did not reach significance in females.

Conversely, percent time freezing during the tone in the cued recall tests was not different between groups. All mice showed the expected increase in freezing when the tone was played at both 24 h (*p* < 0.001; Supplementary Figure 1A) and 2 weeks (*p* < 0.001; Supplementary Figure 1B). There was a time-by-sex interaction at 2 weeks (*p* < 0.001), where females froze less during the baseline period than males. When males and females were analyzed separately, there was a time-by-dose interaction in males only (*p* = 0.043), which appeared to be driven by the saline and 53.5 mg/kg groups showing similar freezing levels during the baseline and tone. No effects were found in females at the 2-week time point.

Hippocampus and amygdala from these animals were assessed for ΔFosB levels by Western blot (Figure 2D). Analysis of hippocampal ΔFosB normalized to total protein revealed that females had higher levels than males (*p* = 0.0029; Figure 2E, *left*). There was also a trend towards an effect of dose (*p* = 0.0521). When sexes were analyzed separately, no effect of dose was seen in males (*p* = 0.6366). Conversely, an effect of dose was seen in females (*p* = 0.0244), with Dunnett’s post hoc test indicating a trend towards a decrease in females that received 107 mg/kg (*p* = 0.0569). Amygdala ΔFosB levels were also higher in females than males (*p* < 0.0001), but were not affected by dose (Figure 2E, *right*).

We next trained a cohort of animals in the same fear conditioning task and immediately delivered an *i.p.* injection of saline or 107 mg/kg of amifostine upon completion (Figure 2F). Injection time (pre- vs. post-training) affected baseline motion, where animals that received pre-training injections moved more at baseline that animals that received post-training injections regardless of drug (*p* < 0.001, Figure 2G). No differences were detected in shock response or percent time freezing during training (Table 1).

When we compared contextual freezing between pre- and post-training injections at 24 h and 2 weeks, a time by sex interaction (*p* = 0.004) and dose by sex interaction (*p* = 0.033) led us to split males and females for analysis. Amifostine increased time freezing in male mice (*p* = 0.015; Figure 2H, *left*) regardless of injection time. Conversely, there were no effects or interactions in females (Figure 2H, *right*).

Analysis of time freezing during the tone in the cued recall tests at 24 h and 2 weeks revealed that females froze more than males (*p* = 0.029) and a trend towards an effect of dose (*p* = 0.088). There were no significant differences based on injection time (*p* = 0.736), and no significant differences were found when we analyzed the sexes separately. All mice showed the expected freezing increase in response to the tone at 24 h (*p* < 0.001; Supplementary Figure 1C) and 2 weeks (*p* < 0.001; Supplementary Figure 1D), though this was distinct between males and females, shown by a time by sex interaction at both time points (*p* = 0.010, *p* < 0.001, respectively). When we split males and females for analysis, we found an effect of injection time in females only at 24 h (*p* = 0.046), where post-training injections led to higher freezing. There were no effects detected during the 2 week recall test (Supplementary Figure 1D).

We also assessed hippocampal ΔFosB in animals that received post-training injections (Figure 2I). When we compared pre- and post-training injections of saline or 107 mg/kg of amifostine in males and females, we found an effect of injection time (*p* = 0.001), dose (*p* = 0.049), and sex (*p* = 0.011), and a dose by time interaction (*p* = 0.045). To clarify these effects, we split males and females. We detected an effect of injection time in males (*p* = 0.041, Figure 2I *left*) and an effect of dose in females (*p* = 0.044, Figure 2I *right*). Post hoc tests did not indicate any differences between specific groups, though these results indicate intriguing sex- and time-dependent effects of amifostine.

### Amifostine decreases hippocampal DSBs but does not change sex-dependent cFos levels

**Figure 3:**
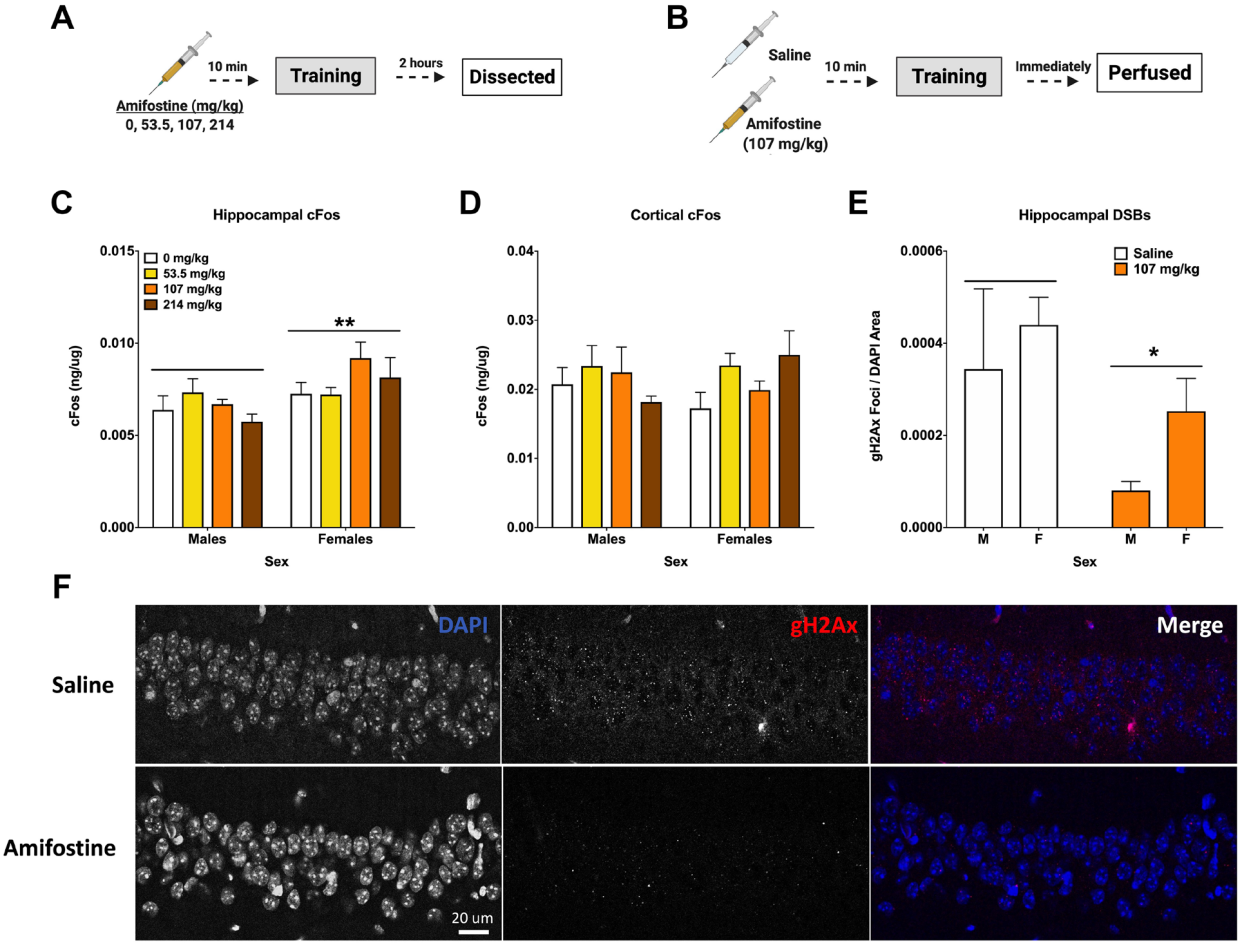
Amifostine decreases hippocampal DSBs but not cFos. (**A**) Experiment 3 design. (**B**) Experiment 4 design. (**C**) Hippocampal cFos levels were measured by ELISA. Females showed a higher levels of hippocampal cFos than males (*F*(1,32) = 8.387, *p* = 0.0068), which was confirmed by Western blot analysis (*F*(1,32) = 6.222, *p* = 0.0180). No effect of dose was detected. (**D**) Cortical cFos levels were measured by ELISA. There were no overall main effects detected, however Dunnett’s post hoc test revealed a trend towards higher cFos in females injected with 214 mg/kg than saline controls (*p* = 0.084). (**E**) Quantification of γH2Ax foci per DAPI area. An injection of 107 mg/kg of amifostine prior to fear training decreased γH2Ax in the CA1 (*F*(1,20) = 5.167, *p* = 0.0342). (**F**) Representative images for γH2Ax IHC of the CA1. Data show averages ± SEM. ^*^ *p* < 0.05; ^**^ *p* < 0.01.

We used a separate cohort of mice to assess the immediate effects of pre-training injections of amifostine on DSBs and IEGs. Hippocampal tissue from animals euthanized 2 hours after training was assessed for cFos, FosB, and NADPH (Figure 3A). Analysis of hippocampal cFos by ELISA indicated that females had higher levels than males (*p* = 0.0068; Figure 3C), which was confirmed with Western blot (*p* = 0.0180, Table 2). No sex or dose differences were detected in hippocampal FosB or NAPDH, nor in γH2Ax when assessed with Western blot (Table 2).

**Table 2:** Molecular measures from mice in experiments 1 and 3

Amifostine				
Dose (mg/kg)	0	53.5	107	214
**Hippocampal FosB (pg/ug)**	0.33 ± 0.006	0.48 ± 0.014	0.40 ± 0.007	0.41 ± 0.007
**Hippocampal NADPH (ug/mg)**	40.99 ± 0.25	46.06 ± 0.21	41.48 ± 0.23	46.97 ± 0.17
**Hippocampal yH2Ax (yH2Ax/Total Protein)**	0.087 ± 0.004	0.0971 ± 0.006	0.0859 ± 0.004	0.091 ± 0.005
**Cortical NADPH (ug/mg)**	35.88 ± 0.33	40.66 ± 0.30	38.11 ± 0.33	31.50 ± 0.25

**Etoposide**			
**Dose (mg/kg)**	**0**	**35**	**70**
**Hippocampal FosB (pg/ug)**	0.302 ± 0.003	0.394 ± 0.006	0.288 ± 0.004
**Hippocampal cFos (ug/mg)**	4.71 ± 0.29	5.06 ± 0.15	5.03 ± 0.37
**Hippocampal yH2Ax (yH2Ax/Total Protein)**	0.116 ± 0.017	0.093 ± 0.010	0.10 ± 0.012
**Cortical NADPH (ng/ug)**	2.92 ± 0.16	2.87 ± 0.13	2.62 ± 0.17

In the amifostine cohorts, there were no differences detected based on dose or sex for hippocampal FosB, NADPH, or γH2Ax, nor in cortical NADPH. In the etoposide cohorts, there were no differences detected based on dose or sex for hippocampal FosB, cFos, or γH2Ax, nor in cortical NADPH. All data are presented as means ± SEM.

No differences based on dose or sex were detected when cortical tissue was analyzed for cFos and NADPH levels; however, Dunnett’s post hoc test indicated a trend towards a difference between 214 mg/kg and 0 mg/kg in females (*p* = 0.084, Figure 3D).

To determine the effects of systemically administered amifostine on DSBs, a cohort of animals received injections of saline or 107 mg/kg of amifostine, were trained in fear conditioning, and euthanized immediately by perfusion for immunohistochemistry (Figure 3B). Analysis of γH2Ax foci in the CA1 region of the hippocampus indicated a decrease in both males and females that received amifostine compared to saline (*p* = 0.0342; Figure 3E). Representative images can be seen in Figure 3F.

### Post-training injections of etoposide decrease contextual and cued freezing in females

**Figure 4:**
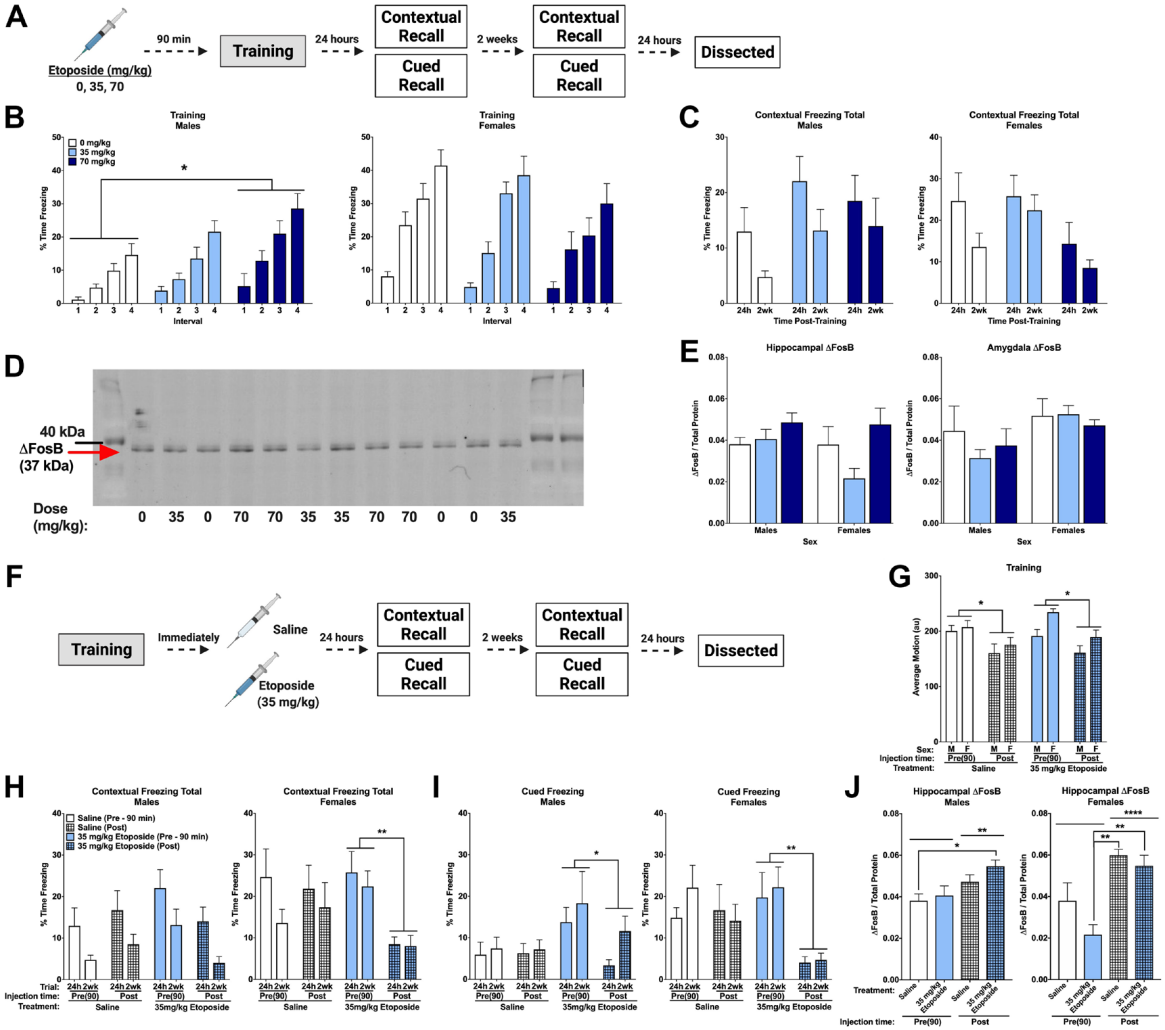
Post-training injections of etoposide lead to long-term decreases in contextual and cued freezing in females. (**A**) Experiment 1 design. (**B**) Percent time freezing during the inter-stimulus intervals on training day. A repeated measures ANOVA in males (*left*) showed a main effect of dose (*F*(2,37) = 4.358, *p* = 0.020) with Dunnett’s post hoc test indicating that males injected with 70 mg/kg froze more than 0 mg/kg (*p* = 0.0106). There was no main effect of dose detected in female mice (*p* = 0.216, *right*). (**C**) Total percent time freezing in males (*left*) and females (*right*) during the 24 h and 2 week contextual trials. No significant differences were detected. (**D**) Representative Western blot for ΔFosB. (**E**) Quantification of hippocampal (left) and amygdala (right) ΔFosB. A 2-way ANOVA of hippocampal levels revealed a significant main effect of dose (*F*(2,18) = 4.143, *p* = 0.0331), though no differences compared to the saline injections were detected with Dunnett’s post hoc test. Analysis of amygdala levels indicated a trend towards females having higher levels than males (*F*(1,19) = 3.806, *p* = 0.0660). (**F**) Experiment 2 design. (**G**) Average baseline motion during the training session. Regardless of dose, pre-training injections increased average baseline motion (*F*(1,73) = 17.919, *p* < 0.001). Males also moved less than females (*F*(1,73) = 7.239, *p* = 0.009). (**H**) Percent time freezing during the 24 h and 2-week contextual recall tests in males (*left*) and females (*right*). In males, we found a trend towards a significant dose by injection time interaction (*F*(1,27) = 4.024, *p* = 0.055), with a trend towards a difference between pre- and post-training injections of 35 mg/kg etoposide (*p* = 0.087). In females, we saw a trend toward a main effect of injection time (*F*(1,25) = 3.317, *p* = 0.089) and a trend towards a dose by injection time interaction (*F*(1,25) = 3.573, *p* = 0.070). Comparison of injection time revealed a difference between pre- and post-injections of 35 mg/kg etoposide (*p* = 0.003). (**I**) Percent time freezing during the tones at 24 h and 2-weeks after training in males (*left*) and females (*right*). Repeated measures analysis in males revealed a significant dose by injection time interaction (*F*(1,27) = 4.292, *p* = 0.048) and a trend towards a main effect of dose (*F*(1,27) = 4.185, *p* = 0.051). Pre- and post-training injections of 35 mg/kg etoposide were significantly different (*p* = 0.035). In females, a significant main effect of injection time (*F*(1,26) = 6.631, *p* = 0.016) and trend toward a dose by injection time interaction (*F*(1,26) = 3.156, *p* = 0.087) were found. A significant difference between pre- and post-training injections of 35 mg/kg etoposide (*p* = 0.007) was found. (**J**) Hippocampal ΔFosB quantification in males (*left)* and females (*right*) that received pre- or post-training injections of saline or 35 mg/kg of etoposide. A 3-way ANOVA showed a significant effect of injection time (*F*(1,37) = 27.412, *p* < 0.0001) and a trend towards a sex by dose (*F*(1,37) = 3.641, *p* = 0.064) and sex by injection time interaction (*F*(1,37) = 3.788, *p* = 0.059). When split, males showed a significant effect of injection time (*F*(1,19) = 9.526, *p* = 0.0061) with post hoc tests indicating a difference between mice injected with saline pre-training compared to mice injected with etoposide post-training (*p* = 0.0359). Females also showed a significant effect of injection time (*F*(1,18) = 26.95, *p* < 0.0001) and a trend towards a dose difference (*F*(1,18) = 4.065, *p* = 0.059). Post hoc tests indicated that pre-training injections of etoposide had lower ΔFosB than post-training saline (*p* = 0.0005) and post-training etoposide (*p* = 0.0020). Data show averages ± SEM. ^*^ *p* < 0.05; ^**^ *p* < 0.01.

We next assessed how pre-training injections of etoposide affect learning, memory, and IEG expression. Mice received *i.p.* injections of saline, 35, or 70 mg/kg of etoposide 90 min before being trained in fear conditioning. Contextual and cued memory were assessed 24 h and 2 weeks later (Figure 4A).

Analysis of the effects of etoposide on learning revealed that females moved more than males during the initial baseline period (*p* = 0.044), though there was no effect of dose. There were no differences during shocks or in percent time freezing during the tones (Table 3). However, percent time freezing during the inter-stimulus intervals was higher in females (*p* = 0.000), and etoposide dose affected freezing based on sex (*p* = 0.010). Males showed an effect of dose (*p* = 0.020), with Dunnett’s post hoc test revealing that mice injected with 70 mg/kg froze more during the intervals than controls (*p* = 0.0106; Figure 4B, *left*). This effect was not detected in females (*p* = 0.216; Figure 4B, *right*), suggesting that pre-training injections of etoposide had sex-dependent effects on learning. Moreover, there were no dose-dependent differences on average motion during the intervals (*p* = 0.872), indicating that etoposide did not affect motion.

**Table 3:** Fear conditioning motion and time freezing during training for mice in experiment 1 and 2 investigating the effects of etoposide

Dose (mg/kg)	Pre-Training Injections			Post-Training Injections
	0	35	70	35
**Baseline Average Motion (au)**	214.16 ± 9.65	210.26 ± 10.52	202.60 ± 10.07	*See Figure 4*
**Shocks Average Motion (au)**	899.24 ± 45.42	797.34 ± 38.86	841.68 ± 39.77	845.82 ± 48.51
**Last Tone % Time Freezing**	28.17 ± 4.59	32.95 ± 5.25	30.02 ± 6.38	30.44 ± 5.15

No differences were detected between sexes or doses. All data are presented as means ± SEM.

There were no differences detected when time freezing during the contextual recall tests was analyzed (Figure 4C). However, minute-by-minute analysis of the 2 week trial revealed a significant effect of dose in females only (*p* = 0.0149; Supplementary Figure 2A), suggesting a possible subtle, female-specific increase. No differences were detected when time freezing during the tone in the cued recall tests at 24 h and 2 weeks was analyzed (Supplementary Figure 2B). All mice showed the expected increase in freezing between baseline and the tone at 24 h (*p* < 0.001; Supplementary Figure 2C) and 2 weeks (*p* < 0.001; Supplementary Figure 2D), with no differences detected based on dose or sex at either time point.

Western blot was used to assess ΔFosB levels in hippocampus and amygdala from these animals (Figure 4D). Analysis of hippocampal ΔFosB revealed an effect of dose (*p* = 0.0331), but no effect of sex and no sex by dose interaction. When we removed sex as a factor from the statistical model, this effect of dose still held (*p* = 0.040), though Dunnett’s post hoc testing did not indicate any differences compared to the saline-injected group (Figure 4E, *left*). Analysis of amygdala ΔFosB by Western blot indicated a trend towards a difference based on sex (*p* = 0.0660), but not dose (Figure 4E, *right*).

We next tested the effects of 35 mg/kg of etoposide administered after training on memory (Figure 4F). As observed before, pre-training injections led to an overall increase in baseline motion compared to post-training injections regardless of drug (*p* < 0.05; Figure 4G). No other differences were detected during training, with all animals showing the expected increase in freezing during training (Table 3). Analysis of percent time freezing during the 24 h and 2 week contextual trials revealed that females froze more than males (*p* = 0.033). When sexes were analyzed separately, we detected a trend towards a drug by injection time interaction (*p* = 0.055), and no effects of drug (*p* = 0.409) or injection time (*p* = 0.445) in male mice (Figure 4H, *left*). In females, we again observed a trend toward a drug by injection time interaction (*p* = 0.070) and a trend towards an effect of injection time (*p* = 0.089). Subsequent pairwise comparison of injection time revealed that females injected with 35 mg/kg of etoposide after training froze significantly less than those that received pre-training etoposide injections (*p* = 0.003; Figure 4H, *right*).

Analysis of freezing during the tone at 24 h and 2 weeks also indicated an effect of sex (*p* = 0.009), an effect of injection time (*p* = 0.002) and an injection time by dose interaction (*p* = 0.010). When we analyzed the sexes separately, we found a trend toward an effect of injection time (*p* = 0.051) and a significant drug by injection time interaction (*p* = 0.048) in males. Post hoc analysis showed that post-training injections in males decreased freezing (*p* = 0.035; Figure 4I, *left*). This was also observed in females (*p* = 0.016), as well as a trend toward an injection by drug interaction (*p* = 0.087). Subsequent analysis indicated that the effect of injection time was driven by the females that received post-training etoposide (*p* = 0.007; Figure 4I, *right*). Assessment of freezing at baseline and during the tone showed that females injected with 35 mg/kg of etoposide after training had a blunted response to the tone at both 24 h (*p* = 0.035) and 2 wks (*p* = 0.004), which was not seen in males (Supplementary Figure 3A–3D).

Hippocampal ΔFosB was also analyzed in these animals compared to pre-training injections at the same doses. We found a significant effect of injection time (*p* < 0.0001) and trends towards a sex by dose interaction (*p* = 0.064) and a sex by time interaction (*p* = 0.059). Analysis in males only showed this same effect of injection time (*p* = 0.0061) but no effect of dose (*p* = 0.199). *Post hoc* comparisons to identify the time differences showed that mice that received saline pre-training had lower hippocampal ΔFosB than 35 mg/kg post-training (*p* = 0.036; Figure 4J *left*). Analysis in females only also showed an effect of time (*p* < 0.0001) and a trend towards an effect of dose (*p* = 0.059); post hoc analysis showed that mice injected with 35 mg/kg etoposide pre-training injections had lower levels than post-training saline (*p* = 0.0005) and post-training 35 mg/kg mice (*p* = 0.002; Figure 4J *right*). Again, these data highlight the importance of sex and timing of administration when assessing effects of etoposide.

### Etoposide decreases hippocampal NADPH in females and hippocampal DSBs in both sexes

**Figure 5:**
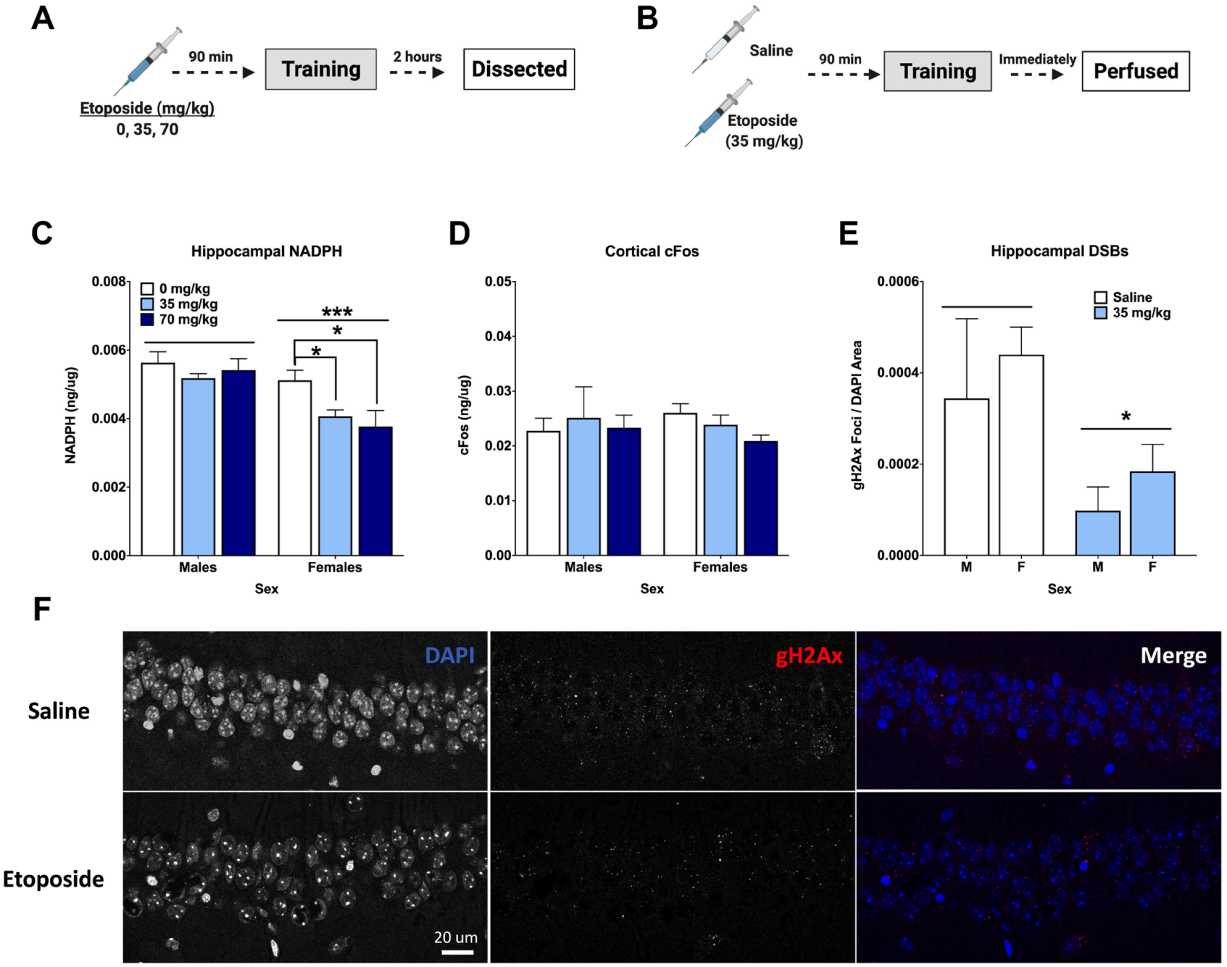
Etoposide decreases hippocampal γH2Ax, hippocampal NADPH, and cortical cFos in a sex-dependent manner. (**A**) Experiment 3 design. (**B**) Experiment 4 design. (**C**) Hippocampal NADPH levels. A 2-way ANOVA showed a main effect of dose (*F*(2,24) = 4.141, *p* = 0.0285) and that males had higher levels than females (*F*(1,24) = 18.68, *p* = 0.0002). A 1-way ANOVA indicated a significant effect in females (*F*(2,12) = 4.404, *p* = 0.0368), with Dunnett’s post hoc test indicating a difference between 0 and 35 mg/kg (*p* = 0.041) and between 0 and 70 mg/kg (*p* = 0.033). There was no effect of dose detected in males (*p* = 0.216). (**D**) Cortical cFos levels. No significant differences were detected by a 2-way ANOVA. In females, a 1-way ANOVA did not reveal an overall difference based on dose (*F*(2,12,) = 2.796, *p* = 0.100), but Dunnett’s post hoc test indicated a trend towards a difference between 0 mg/kg and 70 mg/kg (*p* = 0.0648). There were no differences detected in males by 1-way ANOVA (*p* = 0.8993). (**E**) Quantification of γH2Ax foci per DAPI area. An injection of 35 mg/kg of etoposide prior to fear training decreased γH2Ax in the CA1 (*F*(1,20) = 6.271, *p* = 0.0210). (**F**) Representative images of γH2Ax in the CA1. Data show averages ± SEM. ^*^ *p* < 0.05; ^***^ *p* < 0.001.

We used a separate cohort of animals to assess the immediate effects of pre-training injections of etoposide on DSBs and IEGs. Hippocampal tissue from animals euthanized 2 hours after training was assessed for cFos, FosB, and NADPH (Figure 5A). Males had higher NADPH levels than females (*p* = 0.0002), and NADPH levels were affected by dose (*p* = 0.0285). When we split sexes for analysis, it was clear that this was driven by the females, as an effect of dose was detected in females (*p* = 0.0368), but not males (*p* = 0.526; Figure 5C). Dunnett’s post hoc test indicated that both the 35 mg/kg and 70 mg/kg groups had lower NADPH levels than 0 mg/kg (*p* = 0.041 and *p* = 0.033, respectively). There were no differences detected for cFos, FosB, or γH2Ax when measured by Western blot or ELISAs (Table 2).

Cortical tissue was also analyzed for cFos and NADPH levels. Analysis of cortical cFos levels did not indicate a dose difference in females (*p* = 0.100), but Dunnett’s post hoc test revealed a trend towards a difference between 0 and 70 mg/kg (*p* = 0.0648). This was not seen in males (Figure 5D). Unlike the hippocampus, no differences in cortical NADPH were detected (Table 2).

Another cohort received either saline or 35 mg/kg of etoposide prior to training and were euthanized immediately upon completion (Figure 5B). Analysis of hippocampal DSBs indicated that 35 mg/kg of etoposide decreased γH2Ax foci per DAPI area compared to saline controls in both male and female mice (*p* = 0.0285; Figure 5E). Representative images can be seen in Figure 5F.

## DISCUSSION

To the best of our knowledge, this is the first study to examine the effects of systemic injections of DSB-altering agents commonly used during cancer treatment on learning, memory, and IEG expression in both sexes. Despite their clinical use for decades [24, 25], research into their specific effects is confounded by additional treatments and disease state [18]. Impairments in cognitive function are widely reported among patients undergoing cancer treatment, which is often termed “chemo-brain” [26–29]. Our results suggest that amifostine and etoposide have distinct effects on learning and memory dependent on sex and timing of administration. Both reduce hippocampal γH2Ax signal after fear training, suggesting that interrupting typical DSB formation and repair may lead to disruptions in learning and memory. The middle doses used (107 and 35 mg/kg, respectively), which reflect clinical levels, also had the most pronounced effects on memory.

Amifostine did not impair animals’ ability to learn, but etoposide (70 mg/kg) increased fear learning in males that was not due to effects on motion. Across experiments, we observed sex differences in freezing levels, with females often showing higher freezing than males. This is consistent with previous work showing that female mice froze more during contextual fear conditioning training than males, and showed greater fear generalization to other contexts [30]. This is also reflective of human conditions, as women diagnosed with post-traumatic stress disorder have greater fear acquisition than men [31]. Reports of emotional distress in cancer patients has indicated that women have increased clinically significant emotional distress [32], anxiety, and depression [33] compared to men. While the complexity of cancer makes it nearly impossible to identify a single driving factor, our observation of the sex-dependent effects of these common drugs on fear learning and memory suggest they could be contributing to emotional distress measured in patients.

These compounds also affected long-term memory. We detected an increase in hippocampus-dependent contextual fear memory in male mice injected with 107 mg/kg of amifostine. The similar effects of pre- and post-training amifostine injections is likely due to the relatively fast pharmacokinetics, as amifostine peaks in the brain 7–15 min after systemic administration and we conducted fear training and pre/post injections within a 20 min time window [2]. Conversely, we did not detect any differences in hippocampus-independent cued fear memory, suggesting region-dependent susceptibility. We similarly saw an effect of etoposide on long-term contextual fear memory, though in females and only following post-training injections. On top of that, we also observed a decrease in cued freezing in both sexes after post-training etoposide injections.

Other research on hippocampus-dependent memory following amifostine has suggested similar results. 214 mg/kg of amifostine administered prior to gamma radiation rescued novel object recognition and restored hippocampal neurogenesis in mice [20]. Specific effects of etoposide on hippocampus-dependent and -independent function have not previously been described, even in conjunction with radiation. One study indicated that etoposide may be useful for hippocampal tumors due to reduction in hippocampal polyamines [34], but function was not assessed. Investigations into the effects of combined cancer treatment have identified the hippocampus as particularly susceptible. For example, ovariectomized female rats treated with cyclophosphamide and doxorubicin displayed impaired contextual, but not cued, fear memory 1 week after completing treatment [35]. In patients, hippocampal connectivity was shown to be altered after chemotherapy [27] and reductions in hippocampal volume were correlated with impaired cognition [36].

A possible mechanism contributing to changes in cognition may be IEG regulation. While our data are inconclusive, patterns indicate that that these drugs may affect synaptic plasticity via disrupting IEGs which should be further explored. Here, cortical cFos was mildly affected by both drugs in a sex-dependent manner. Females treated with the highest dose of amifostine trended toward higher cortical cFos levels, whereas females treated with the highest dose of etoposide trended toward lower levels. Additionally, our data suggested that 107 mg/kg lowered hippocampal ΔFosB levels in females, and that etoposide had an overall effect on hippocampal ΔFosB, where 35 mg/kg etoposide group appears to have slightly lower levels.

The complexity of measuring IEG induction and expression is well-known, and observed here in the stark differences in hippocampal ΔFosB levels based on timing of injection (pre-training or post-training). We observed higher levels in most animals that received post-training injections, with the exception of females that received amifostine. The complexity of the IEG expression timeline has been a recent interest in development of cancer treatments [37]. Our results indicate that more work needs to be done for a better understanding of the role of IEGs in cancers and cancer treatments.

Notably, we observed sex differences in our molecular measures. Levels of hippocampal cFos, ΔFosB, and amygdala ΔFosB were higher in females than males, though this was only seen in the amifostine cohorts. Timing of the *i.p.* injections (10 vs. 90 min pre-training) likely has an influence on stress response and IEG levels [38–40]. Other studies suggest that sex differences in cFos activation might be dependent on brain region: male mice had greater dorsal hippocampal cFos induction following contextual fear retrieval, whereas females showed greater basal amygdala induction [30]. Assessment of estrous cycle in rats suggested that sex differences in cFos mRNA were dependent on stage of the cycle [41], which could account for discrepancies in our results and the literature.

Hippocampal NADPH levels were different based on sex in the etoposide cohorts, with higher levels seen in males. Females generally appear to have less oxidative damage than males, resulting in less NAPDH oxidase activity [42]. We also detected a dose-dependent decrease in hippocampal NADPH levels in females. NADPH has previously been shown to counteract the effects of etoposide [43], which is in line with our results, as NADPH levels would decrease as it is oxidized to NADP+. Conversely, NADPH levels were not different based on amifostine dose, which is itself a reactive oxygen species scavenger. These sex-dependent changes in cFos, ΔFosB, and NADPH did not directly mirror the observed behavioral changes. However, our observations are important to consider for future efforts looking at learning, memory, and response to drugs in males and females, as highlighted by Shansky and Murphy [44].

In summary, our results reveal important information about the effects of amifostine and etoposide on learning, memory, and IEGs that can optimize treatment strategies. Newer analogs of these drugs, such as PrC-210 [45], might reduce these side effects and improve patients’ quality of life. Additionally, assessing the direct effects of these drugs on specific brain regions (via central injections) will be useful to clarify the underlying mechanisms driving learning and memory changes. Future investigations are warranted to determine the role of DSBs in encoding, retrieval, and reconsolidation, and further our understanding of learning and memory processes in health and disease.

## MATERIALS AND METHODS

### Mice

Male and female C57Bl/6J (WT) mice (*n* = 299) purchased from Jackson Labs (Sacramento, CA) were used. Mice were 3–4 months old at the time of behavioral testing. All mice were group housed to 5/cage; starting 5 days before behavioral testing, mice were singly housed and provided with extra enrichment. Standard chow and water were provided *ad libitum*. Lights were on a 12-hour cycle; testing took place during the light cycle. All procedures were reviewed and approved by the Institutional Animal Care and Use Committee (IACUC) at the AAALAC-certified Oregon Health and Science University (OHSU).

### Compounds

Amifostine trihydrate (WR-2721) was purchased from Sigma Aldrich (Cat #1019406-150 MG) and diluted in saline solution the day prior to injection. Mice received a single intra-peritoneal (*i.p.*) injection of one of six doses of amifostine: 0 (saline), 53.5, 80.25, 107, 160.5, or 214 mg/kg. These doses were chosen based on a range of typical doses tested in the context of radioprotection [3, 46].

Etoposide phosphate (etopophos) powder was purchased through the OHSU research pharmacy (E.R. Squibb & Sons, LLC, New Brunswick, NJ, USA). It was diluted in saline to a 20 mg/mL stock solution and stored at –80°C until the day before use. Mice received a single *i.p.* injection of etoposide (0, 35, or 70 mg/kg). These doses were chosen based on the reported range commonly used for acute dosing [47].

### Behavioral experiments

#### Experiment 1: effects of pre-training injections on long-term memory

Amifostine was delivered *i.p.* (6 doses, *n* = 8–12 mice/sex/dose) 10 minutes before being placed into a sound-attenuating chamber for fear conditioning (Med Associates, Fairfax, VT). The timing between injection and training was chosen as amifostine peaks in brain tissue 7–15 min after a systemic injection [48]. A separate series of experiments were conducted to test the effects of etoposide. Etoposide was administered *i.p.* 90 min before fear training (3 doses, *n* = 8–9 mice/sex/dose). This route and time of injection were chosen after we assessed concentration of etoposide in the brain following *i.p.* or *i.v.* injection: as both routes showed similar brain levels 2 hours after injection, we proceeded with *i.p.* injections (data not shown).

We followed a similar fear conditioning paradigm as previously described [49]. Mice explored the box for a 2-minute baseline period, after which a 30-second tone (80 dB, 2800 Hz) played that co-terminated with a 2-s foot shock (0.5 mA); this was followed by a 90-s inter-stimulus interval (ISI). The tone-shock pairings were repeated a total of 4 times. All chambers were cleaned with 0.5% acetic acid between trials. The researcher doing the injections and the researcher handling the mice for behavior were distinct, and both were blinded to treatment groups during testing.

To assess long-term hippocampus-dependent and hippocampus-independent memory, mice underwent contextual and cued fear recall tests 24 h and 2 weeks after training as described [19, 23, 49, 50]. For the contextual recall test (hippocampus-dependent), mice were placed into the same chamber for a period of 5 minutes; no tones and no shocks were administered. Chambers were cleaned with 0.5% acetic acid between contextual trials [19, 49]. For the cued recall test (hippocampus-independent), a distinct floor, ceiling, and smell were placed in the chambers. After a baseline period of 90 s, the tone was played for a 3-min period. Chambers were cleaned with 10% isopropanol between cued trials [50].

Twenty-four hours after the last recall test, mice were euthanized by rapid cervical dislocation. Brain tissue was collected: the amygdala and cortex were dissected from half of the animals, while the hippocampus and cortex were dissected from the other half to assess tissue for long-term changes in ΔFosB levels. Tissues were flash frozen in liquid nitrogen and stored at −80°C until use. An experimental timeline is illustrated in Figure 1A (made with https://biorender.com).

#### Experiment 2: effects of post-training injections on long-term memory

To ensure that differences in long-term memory were not a result of differences induced by either drug during acquisition, we injected animals immediately after fear training. Based on the results of experiment 1, we chose a single dose of amifostine (107 mg/kg) and a single dose of etoposide (35 mg/kg) to compare to post-training saline injections for these experiments (*n* = 7/dose/sex). Fear training, contextual, and cued recall tests were all performed in the same way as described above (Figure 1B).

#### Experiment 3: effects of pre-training injections on cFos and NADPH

To assess the effects of amifostine and etoposide on IEG expression, mice were euthanized by cervical dislocation 2 hours after systemic injection followed by fear training to capture peak cFos signal [10]. For this experiment, we used 4 doses of amifostine (*n* = 5 mice/sex/dose: 0 (saline), 53.5, 107, or 214 mg/kg) or 3 doses of etoposide (*n* = 5 mice/sex/dose: 0 (saline), 35, or 70 mg/kg). Hippocampus and cortex were dissected from all animals and flash frozen in liquid nitrogen. Tissue was stored at −80°C until use (Figure 1C).

#### Experiment 4: effects of pre-training injections on DSBs

To assess the effects of amifostine on DSB formation in the hippocampus, mice were injected with saline, 107 mg/kg of amifostine, or 35 mg/kg of etoposide and euthanized immediately upon completion of fear conditioning via perfusion (Figure 1D).

### Western blots

Brain tissue was prepared as described [51]. Tissue was homogenized and sonicated in lysate buffer (1M Tris-Cl, pH 7.5; 6M NaCl; 10% SDS; 0.5M EDTA; Triton-X 100; Phosphatase Inhibitor #3, Roche, #05-892-970-001; Protease Inhibitor, Sigma-Aldrich, #P0044) and total protein concentrations determined with a Pierce BCA Kit (Thermo Fisher, #23227).

Samples were boiled in SDS buffer for 10 min and 10 μg of protein loaded into wells of 10–20% Tris-Glycine gels. Gels were run for 75 min at 125 V to separate samples. Proteins were transferred to Immobilon-FL PVDF membranes (Millipore, #IPF00010) for 75 min at 30 V on ice. Total protein for quantification was assessed using REVERT^™^ Total Protein Stain (Li-Cor Biosciences, Lincoln, NE, USA) and imaged on a Li-Cor Odyssey CLx. Blots were then blocked in Odyssey Blocking Buffer (Li-Cor Biosciences) for 1 h at room temperature and incubated overnight at 4°C with primary antibodies against ΔFosB (Cell Signaling, Cat# 14695S, rabbit, 1:1000), cFos (Santa Cruz, Cat #2119, mouse, 1:500), or γH2Ax (Cell Signaling, Cat #9718, rabbit, 1:1000,). The following day, blots were washed and incubated in appropriate secondary antibodies (goat anti-rabbit IR800 CW, Li-Cor, #926-32211, 1:10,000; goat anti-mouse IR700 IR680 LT, Licor, #926-68050, 1:10,000) for 1 h at room temperature.

Images were analyzed with ImageJ software and normalized to total protein detected by the REVERT stain for comparison. More details can be found in Supplementary Materials.

### ELISAs

cFos, FosB, and NADPH levels were analyzed using ELISA kits from MyBioSource (San Diego, CA, USA) according to manufacturer directions. Hippocampus and cortical samples from experiment 3 were assessed for cFos (Cat# MBS2887418; HIP 1:10, CTX 1:100) and NADPH (Cat# MBS2605848; AMY 1:10, HIP 1:20, CTX 1:40); hippocampus samples were also assessed for FosB (Cat# MBS9718096; HIP 1:10). Hippocampus and amygdala samples from experiment 1 were analyzed for FosB and NADPH levels. Standards were run in duplicate on all plates; samples were run singly. For analysis, all optical density readings were normalized to total protein determined by a Pierce BCA Kit (Thermo Fisher, #23227).

### Immunofluorescence & microscopy

Perfused brains from experiment 4 were cut into 40 μm sections using a Cryostat to assess the formation of γH2Ax foci, a common marker for DSB repair [6, 7]. We followed our standard immunofluorescent protocol [51]. Briefly, sections were rinsed in 1x PBS and blocked in 4% normal goat serum before overnight incubation in primary antibody targeting γH2Ax (rabbit-anti-γH2Ax, Cell Signaling, #9718, 1:500). On day 2, sections were rinsed and incubated in secondary antibody (goat-anti-rabbit, AlexaFluor594, Invitrogen, #A-11012, 1:250) for 2 hours. Following washes, 2.5 μg/mL DAPI (Sigma D9542) in PBS was applied for 20 min. Sections were rinsed one more time, then slide mounted with CitiFluor CFMR2 Antifadent Solution, and sealed with Biotium CoverGrip Coverslip Sealant. Z-stack images at 0.5 μm steps were taken using a Zeiss LSM 980 with Airyscan 2 at 63× zoom.

ImageJ (NIH) was used to analyze γH2Ax foci. ROIs were created for each nucleus in the DAPI channel based on a single image in the center of the Z-stack. Within the mask, the number and size of γH2Ax foci were quantified.

### Statistical analysis

Researchers were blinded throughout all experiments. Data were assessed for normality of variance. Statistical analyses were performed using SPSS v.25 software (IBM, Armonk, NY, USA) or GraphPad v.7 software (Prism, San Diego, CA, USA). Analyses for effects of amifostine and etoposide were performed separately. Sex and dose were used as between-group variables; when sex was not found to be significant, it was dropped from the statistical model. When sex was found significant, analysis of the sexes was done separately. Dunnett’s *post hoc* test was used to compare doses against saline following all ANOVAs, as statisticians indicate that running *post hoc* tests in the absence of significant ANOVAs is informative and valid [52].

#### Fear conditioning

For analysis of training, data from all experiments with pre-training injections of amifostine were combined, and data from experiments with pre-training injections of etoposide were combined. Average motion and percent time freezing were analyzed using Analysis of Variance (ANOVAs). Total percent time freezing during the 24 h and 2 week contextual tests was analyzed with a repeated measures ANOVA; additionally, time freezing over the 5 minutes within each trial was analyzed with a repeated measures ANOVA. For the cued recall tests, percent time freezing during the tone at 24 h and 2 weeks was analyzed with a repeated measures ANOVA. A repeated measures ANOVA was also used to analyze percent time freezing during the baseline and tone periods for each recall test.

To assess the effects of pre- vs. post-training injections on contextual and cued recall, the corresponding doses of amifostine and etoposide from experiment 1 were analyzed against the 24 h and 2 week contextual and cued recall tests from experiment 2 with ANOVAs.

#### Molecular measures

For analysis of Western blots, bands of interest were normalized to total protein transferred to the blot and analyzed with a 2-way ANOVA. For analysis of ELISAs, optical density readings were interpolated based on the standard curve in each plate using GraphPad v. 7 and normalized to total protein determined with the BCA kit. For analysis of γH2Ax foci, the size and count of foci were normalized to total DAPI area. A 2-way ANOVA was used to analyze differences based on dose and sex. In the case of experiments 2 and 4, the same saline control group was used for comparison against amifostine an etoposide respectively, with corrections for type I statistical errors applied.

## SUPPLEMENTARY MATERIALS



## Abbreviations

DSBs: Double strand breaks
IEGs: immediate early genes
*i.p.*: intraperitoneal
NADPH: Nicotinamide adenine dinucleotide phosphate
