# Supplementary material for: Common cancer treatments targeting DNA double strand breaks affect long-term memory and relate to immediate early gene expression in a sex-dependent manner
Source: Oncotarget. 2022 Jan 24;13:198–213. doi: 10.18632/oncotarget.28180 (PMC8794536; doi:10.18632/oncotarget.28180)
Supplement: Supplementary file 1 [file oncotarget-13-28180-s001.pdf]

Common cancer treatments targeting DNA double strand breaks affect long-term memory and relate to immediate early gene expression in a sex-dependent manner

SUPPLEMENTARY MATERIALS

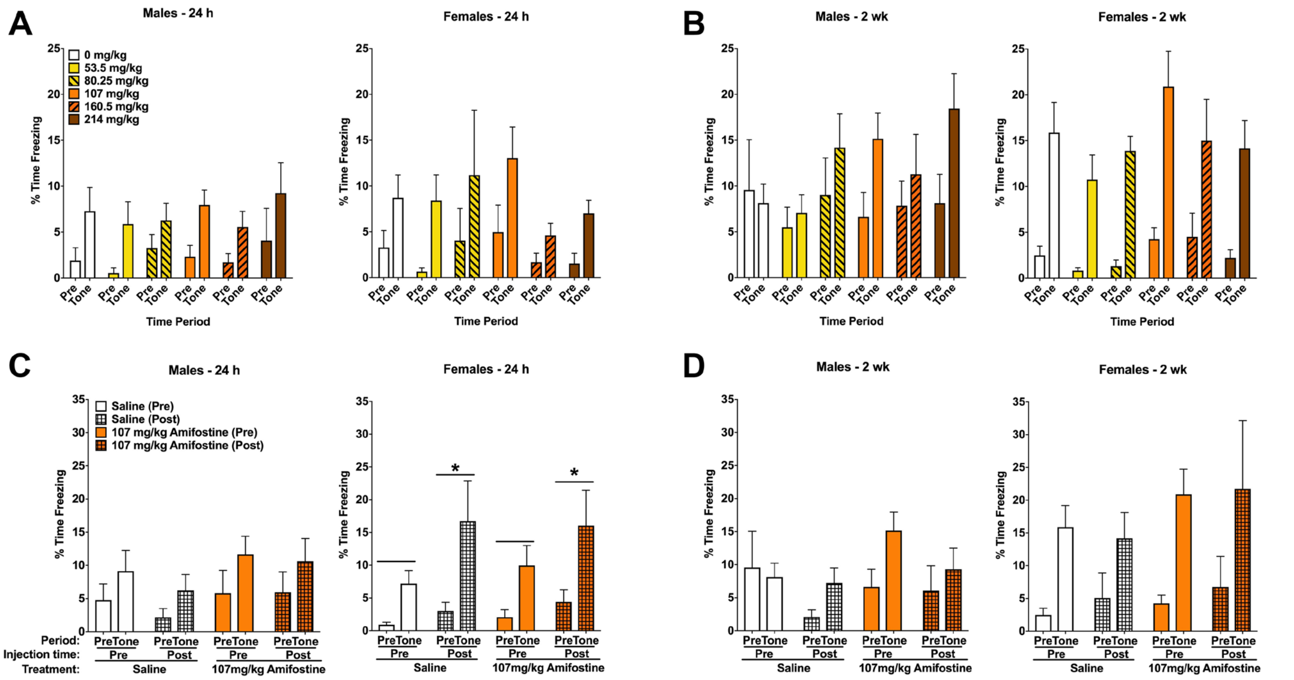

Supplementary Figure 1: Cued recall tests at 24 h and 2 wks after pre- and post-training injections of amifostine. (**A**) Percent time freezing at baseline ("pre") and in response to the tone 24 h after pre-training injections in males (*left*) and females (*right*). All groups froze more in response to the tone ($F(1,108) = 77.189$, $p < 0.001$). (**B**) Percent time freezing at baseline and during the tone 2 wks after pre-training injections in males (*left*) and females (*right*). All groups froze more when the tone played ($F(1,108) = 99.639$, $p < 0.001$). We also found a significant time by sex interaction ($F(1,108) = 21.281$, $p < 0.001$), with females freezing less during the baseline period. Analysis in males showed a significant time by dose interaction ($F(5,54) = 2.472$, $p = 0.043$). (**C**) Percent time freezing at baseline and during the tone 24 h after post-training injections in males (*left*) and females (*right*). All groups showed an increase in response to the tone ($p < 0.001$), though we found a time by sex interaction ($F(1,64) = 6.961$, $p = 0.010$). Analysis in females revealed a significant effect of injection time ($F(1,34) = 4.270$, $p = 0.046$). (**D**) Percent time freezing at baseline and during the tone 2 wks after post-training injections in males (*left*) and females (*right*). All groups showed increased freezing in response to the tone ($F(1,64) = 42.424$, $p < 0.001$). We also found a time by sex interaction ($F(1,34) = 14.952$, $p = 0.000$). Data show averages ± SEM. $^{*}p < 0.05$.

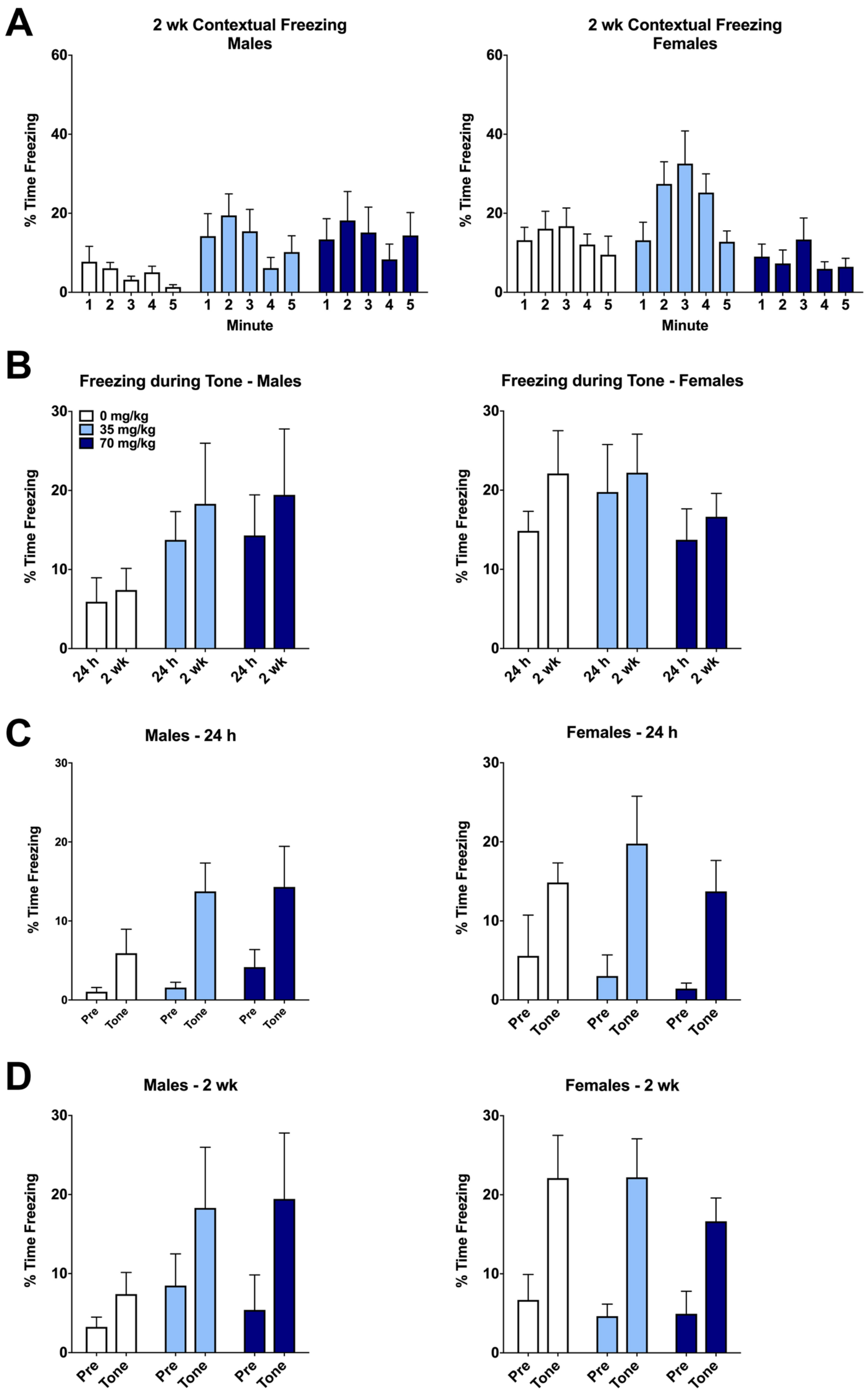

Supplementary Figure 2: Cued recall tests at 24 h and 2 wks after pre-training injections of etoposide. (**A**) Percent time freezing over the 5 minutes of the 2 wk contextual recall test in males (*left*) and females (*right*). Minute-by-minute analysis indicated a main effect of dose in females ($F(2,21) = 5.173$, $p = 0.0149$). (**B**) Percent time freezing during the tone in the 24 h and 2 wk recall tests in males (*left*) and females (*right*). No differences based on dose or sex were detected. (**C**) Percent time freezing at baseline ("pre") and during the tone in the 24 h recall tests in males (*left*) and females (*right*). All mice showed the expected increase in freezing in response to the tone ($F(1,43) = 54.258$, $p < 0.001$). (**D**) Percent time freezing at baseline and during the tone in the 2 wk recall tests in males (*left*) and females (*right*). All mice showed the expected increase in freezing in response to the tone ($F(1,43) = 66.264$, $p < 0.001$). Data show averages ± SEM. $^{*}p < 0.05$.

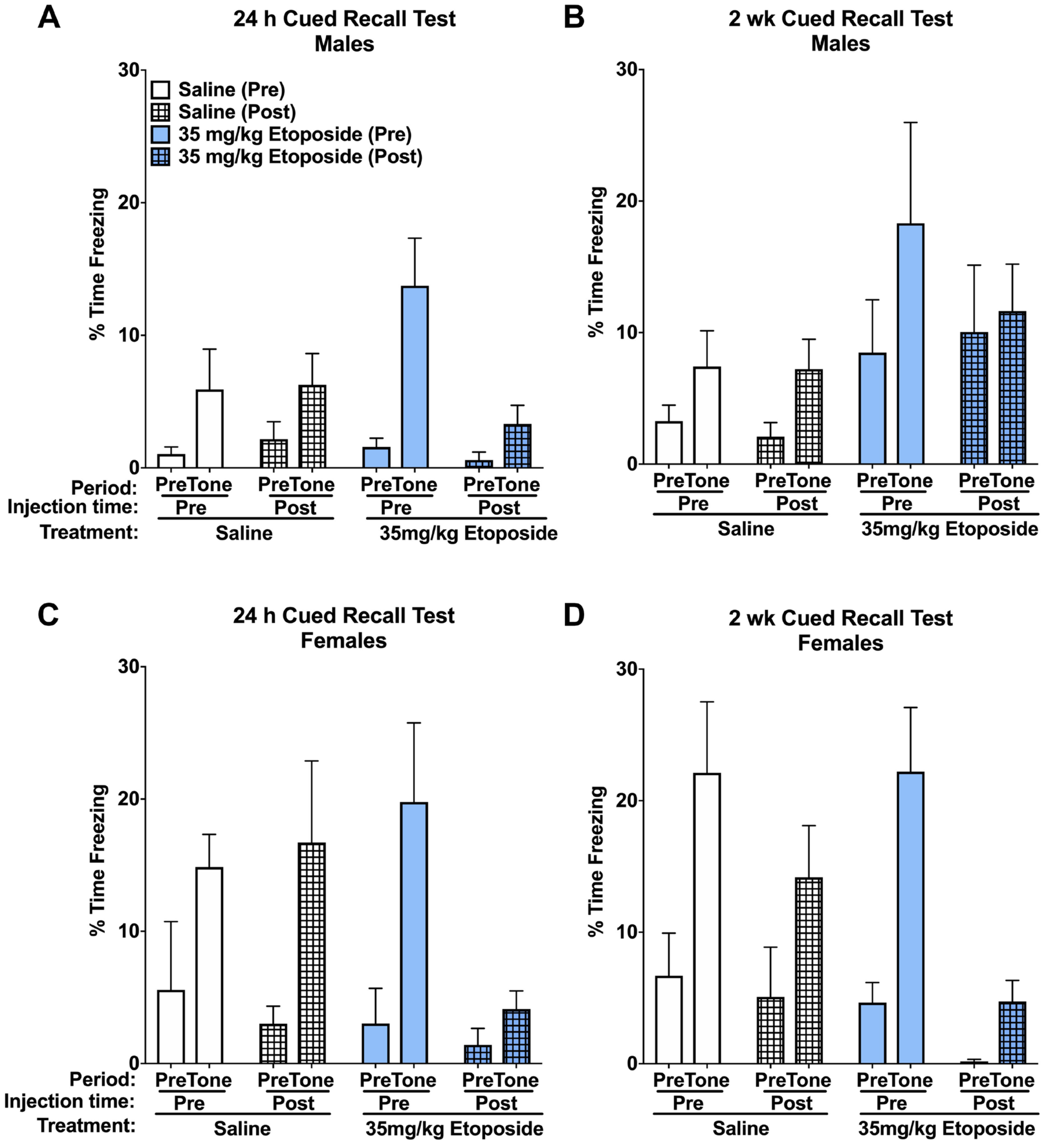

Supplementary Figure 3: Cued recall tests at 24 h and 2 wks after post-training injections of etoposide. (**A**) Percent time freezing at baseline ("pre") and during the tone in the 24 h recall tests in males. All mice increased freezing during the tone ($F(1,27) = 20.733$, $p < 0.001$). (**B**) Percent time freezing at baseline and during the tone in the 2 wk recall tests in males. All mice showed the expected increase in response to the tone ($F(1,27) = 18.359$, $p < 0.001$). (**C**) Percent time freezing at baseline and during the tone in the 24 h recall tests in females. All mice increased freezing in response to the tone ($F(1,26) = 26.036$, $p < 0.001$), but a time by injection time by dose interaction indicated a blunted response in females that received post-training injections of etoposide ($F(1,26) = 4.935$, $p = 0.035$). (**D**) Percent time freezing at baseline and during the tone in the 2 wk recall tests in females. All mice showed an increase in response to the tone ($F(1,26) = 60.079$, $p < 0.001$), though a time by injection time interaction indicated a blunted response in females that received post-training injections of 35 mg/kg etoposide ($F(1,26) = 10.307$, $p = 0.004$).
